# Complete Genome Sequence of a *Pseudomonas* Species Isolated from Tailings Pond Water in Alberta, Canada

**DOI:** 10.1128/MRA.01174-20

**Published:** 2021-03-04

**Authors:** Steven Shideler, John Headley, Jeff Gauthier, Irena Kukavica-Ibrulj, Roger C. Levesque, Shawn Lewenza

**Affiliations:** a Department of Microbiology, Immunology and Infectious Diseases, University of Calgary, Calgary, Alberta, Canada; b Environment and Climate Change Canada, National Hydrology Research Centre, Saskatoon, Saskatchewan, Canada; c Institute of Integrative Biology and Systems (IBIS), Université Laval, Laval, Quebec, Canada; d Faculty of Science and Technology, Athabasca University, Athabasca, Alberta, Canada; Loyola University Chicago

## Abstract

We report the complete genome sequence of strain OST1909, belonging to a *Pseudomonas* species. The genome size is 6,306,352 bp, with a G+C content of 59.6%. The isolate was recovered from oil sands process-affected water (OSPW), despite the numerous toxic compounds that accumulate in oil sands tailings ponds.

## ANNOUNCEMENT

Tailings ponds are large reservoirs of oil sands process-affected water (OSPW), the product remaining after the extraction of heavy oil from mined bitumen, which contains polycyclic aromatic hydrocarbons, BTEX (benzene, toluene, ethyl benzene, and xylenes), and heavy metals (1). Understanding the survival mechanisms of microbes from OSPW may prove useful in providing bioremediation strategies for treating OSPW. *Pseudomonas* sp. strain OST1909 was isolated by plating OSPW samples (from an operator in the Athabasca oil sands region) onto *Pseudomonas* isolation agar and incubating them at room temperature. Single colonies were purified and used for DNA isolation and long-term storage.

Genomic DNA was purified from *Pseudomonas* isolation agar (BD, Sparks, MD)-grown cultures using the DNeasy blood and tissue kit (Qiagen, Hilden, Germany) and mechanically fragmented for 40 s using a Covaris M220 ultrasonicator (Woburn, MA), and library synthesis was performed with the KAPA HyperPrep kit (Kapa Biosystems, Wilmington, MA). TruSeq HT adapters (Illumina, San Diego, CA) were used to barcode the library. Short reads were obtained using an Illumina MiSeq 2 × 300-bp paired-end run, as part of the International *Pseudomonas* Consortium Database (https://ipcd.ibis.ulaval.ca) (2).

Long sequencing reads were obtained using the Oxford Nanopore Technologies MinION platform (R9.4.1 flow cell). DNA was extracted from LB-grown cultures using a standard phenol-chloroform-isoamyl extraction method. A ligation sequencing kit (SQK-LSK109) was used for MinION library preparation. MinION sequencing was performed with MinKNOW v.19.12.2. The raw sequencing data (fast5 format) were base called postsequencing using Guppy v.3.4.1, and final demultiplexing was performed using qcat v.1.1.0 (3).

FastQC v.0.11.9 (4) and Cutadapt v.2.8 (5) were used to filter 1,221,550 raw Illumina short reads and remove adapters, resulting in 1,069,133 paired-end reads, with a mean length of 260 bp, providing 46× sequencing coverage. Filtlong v.0.2.0 (https://github.com/rrwick/Filtlong) was used to quality control the MinION long-read sequences using the filtered Illumina short reads as a reference. Filtlong selected sequence reads that were >1,000 bp long, with a >85% identity match to the Illumina short reads. Out of 362,967 raw long reads, Filtlong selected 149,960 sequences with an average length of 5,335 bp and an *N*_50_ value of 7,054 bp, providing 127× sequencing coverage. Using the Unicycler assembly pipeline v.0.4.8 (6), a hybrid genome assembly was performed using the short- and long-read sequences. A complete, single-contig, circularized genome sequence was assembled, with a size of 6,306,352 bp and a G+C content of 59.62% (QUAST v.5.0.2) (7). The publicly available genome sequence was annotated using PGAP v.4.13 (8).

To identify this organism, we performed whole-genome *in silico* digital DNA-DNA hybridization (dDDH) using the Type Strain Genome Server (TYGS) pipeline (9). The strain Pseudomonas paralactis DSM 29164 (GenBank accession number JYLN00000000) was the closest match based on whole-genome comparison. A dDDH value of 52.4% (confidence interval, 49.7% to 55.1%) between *P. paralactis* DSM 29164 and OST1909 is less than the 70% species threshold and indicates that OST1909 is a closely related species of *Pseudomonas*. Phylogenetic analyses with 16S rRNA and whole-genome sequences from the TYGS position this strain within the Pseudomonas fluorescens group of the P. fluorescens complex (10).

### Data availability.

The genome sequences and raw data have been deposited in GenBank under BioProject PRJNA325248 and BioSample SAMN16313330. The genome accession number is CP063780, and the SRA accession numbers are SRR12781617 (Illumina) and SRR12825871 (Nanopore).
